# Bioactivity Guided Fractionation and Elucidation of Anti-Cancer Properties of *Imperata* *Cylindrica* Leaf Extracts

**DOI:** 10.31557/APJCP.2020.21.3.707

**Published:** 2020-03

**Authors:** Rohini Keshava, Nagesh Muniyappa, Rajalakshmi Gope

**Affiliations:** 1 *Department of Biotechnology, Faculty of Life and Allied Health Sciences, Ramaiah University of Applied Sciences, University House, Gnanagangothri Campus, New BEL Road, MSR Nagar, *; 2 *Strand Lifescience Pvt. Ltd, *; 3 *Department of Human Genetics, NIMHANS, Bangalore, Karnataka, India. *

**Keywords:** Oral cancer, SCC-9, Imperata cylindrical, anticancer, apoptosis, Caspase

## Abstract

**Background:**

In our earlier study, we reported the anticancer effect of methanolic extracts of, *I. cylindrica* leaf (ICL) against human oral squamous cell carcinoma cell lines SCC-9. The cytotoxic effect of ICL methanolic extract was specific to the cancer cells and not to the normal cells. The present study aimed to fractionate the ICL methanolic extract to derive anticancer bioactives.

**Methods:**

The ICL methanolic extract was subjected to a bioactivity guided fractionation. Cytotoxic, cell cycle inhibitory, apoptosis and caspase gene expression inducing activity of the active fractions were evaluated using MTT assay, FACS analysis, Annexin V binding assay and RT-PCR respectively.

**Results:**

The hexane fraction of ICL methanolic extract (ICLH) was observed to be the most bioactive fraction. It was shown to possess effective cytotoxic and cell cycle inhibitory activities against SCC-9 cells. The hexane fraction also induced apoptosis in SCC-9 cells which was further established at the level of caspase 3 and 8 gene expressions.

**Conclusion:**

Overall, the results clearly establish the potential of ICLH extract to inhibit cell proliferation and induce apoptosis in the SCC-9 cells. Further analysis of the ICLH fraction could result in development of effective anticancer therapeutics. The natural abundance of *I. cylindrica* with its wide geographic distribution could make it a preferred natural resource for obtaining novel, cost-effective, anticancer therapeutics with minimal systemic side effects.

## Introduction

Cancer affects human population globally. There is a constant demand for new cost-effective therapies to treat, manage and possibly cure this life-threatening disease (Greenwell and Rahman, 2015). Oral cancer (OC) is reported as the sixth most common cancer globally with males showing a higher incidence and mortality than females (Coelho 2012). OC is a major problem in the Indian sub-continent, and is of significant public health concern (Coelho 2012). Oral squamous cell carcinoma (OSCC) is the most prevalent OC (Ghantous et al., 2015). The survival outcomes of patients have not considerably improved despite recent advancements in therapeutic approaches (Nobrega et al., 2018). 

Although conventional cancer treatments such as surgery, chemotherapy and radiation therapy are beneficial, often times they exert severe, systemic side effects to the patients affecting their quality of life post treatment (Qi et al., 2010). In addition, emergence of resistant cancer cells post therapeutic interventions has necessitated the development of alternative approaches to treat/manage OC. In this regard, the scientific community has drawn its attention towards naturally-derived compounds as they are considered to have less toxic side effects compared to chemotherapy. Plant derived compounds have often demonstrated their abilities to inhibit cancer cell survival by inhibiting cell proliferation and inducing apoptotic cell death (Greenwell and Rahman, 2015). In general, naturally derived compounds from plants are well tolerated and they are non-toxic to normal human cells (Unnati et al., 2013). 

*Imperata cylindrica* Raeusch, (Poaceace), a commonly available grass species, has been known to have several medicinal applications and has been shown to have anticancer effect on several cell lines (Kuete et al., 2011; Kuete et al., 2013; Kwok et al., 2016, Wang et al., 2018; Ravi et al., 2018). Previously we reported the anticancer effect of the methanolic extracts of, *I. cylindrica* leaf (ICL) against human oral squamous cell carcinoma cell line SCC-9. We have shown that the ICL methanolic extracts inhibited cell proliferation and induced apoptosis in the SCC-9 cells. In addition, we found that the cytotoxic effect of ICL methanolic extract was specific to the cancer cells and they had negligible effect on the normal cells (Keshava et al., 2016). In continuation with our previous study, the present study was aimed at obtaining the anticancer bioactive components of the ICL methanolic extract. Experiments were conducted to fractionate the ICL methanolic extract to obtain the bioactive fractions. These fractions were further evaluated for their cell cycle inhibitory and apoptosis inducing properties against the human oral cancer cell line SCC-9. 

## Materials and Methods

*Chemicals and reagents*

Tris-HCl, NaCl, EDTA, SDS, Triton X-100, dimethyl sulfoxide (DMSO), formaldehyde and, DNase free RNase -A were purchased from Merck Co. Mumbai, India. Dulbecco’s Modified Eagles Medium (DMEM), fetal bovine serum (FBS), penicillin-streptomycin and trypsin-EDTA were purchased from Gibco BRL (Life technologies, Grand Island, NY, USA). 3-(4, 5-Dimethylthiazol-2-yl)-2,5 Diphenyltetrazolium Bromide (MTT) reagent was obtained from Sisco Research Laboratories Pvt. Ltd., Mumbai, India. Annexin V-FITC Apoptosis Detection Kit was obtained by Sigma-Aldrich, India. Propidium iodide (PI) was obtained from Beckton Dickinson, Gurgaon, Haryana, India. The primers for the RT-PCR were obtained from Euro fins Genomics, India.

*Cell lines and culture conditions*

Human tongue squamous cell carcinoma cell line, SCC-9 (ATCC CRL-1629) was obtained from American Type Culture Collection (ATCC, Manassas, VA, USA) and maintained as recommended by the supplier. The SCC-9 cells were grown in DMEM medium supplemented with 2.5 mM L-glutamine, 15 mM HEPES, and 10% FBS, along with 100 U/ml penicillin and 100 µg/ml streptomycin. These cells were maintained at 37ºC in a humidified incubator under 95% air atmosphere plus 5% CO_2_ (Keshava et al., 2016).

*Plant material collection and authentication*

The *Imperata cylindrica* plant was collected, the herbarium specimen was authenticated at the botanical garden, University of Agricultural Sciences, G.K.V.K campus, Bangalore, Karnataka, India. A voucher specimen, with collection number - RKDSKPDF01 and accession number - UASB3843, has been deposited at the herbarium, (Keshava et al., 2016). 

*Preparation of Imperata cylindrica leaf (ICL) methanolic extract*

Freshly collected *I. cylindrica* leaves were processed and methanol extract was prepared as described in Keshava et al., 2016. The crude extracts were filtered using Whatman No.1 filter paper (Merck Co. Mumbai, India.) and concentrated using a rotary evaporator (Rotavapor R-100, BUCHI India Pvt. Ltd., Mumbai, India). (Keshava et al., 2016). 

*Liquid-liquid Fractionation of ICL methanolic extract*

A liquid-liquid partition of the ICL methanolic extract was performed. The ICL methanol extract was extracted successively with solvents of increasing polarity. To prepare the fractions, 10 ml of the methanolic extract was transferred to a separatory funnel. The non-polar compounds of the extract were extracted using n-hexane. The extraction was further carried out sequentially with chloroform and methanol to derive semi polar and polar compounds respectively (Shahraki et al., 2015). The, n-hexane (ICLH), chloroform (ICLC) and methanol (ICLM) fractions obtained were further concentrated using a rotary evaporator. 

*Treatment of SCC-9 cells with ICL fractions*

Stock solution of the fractions were prepared by dissolving the concentrated residue in appropriate volume of 100% dimethyl sulfoxide (DMSO) to a final concentration of 64mg/ml, from which required dilutions were prepared. Culture medium was used to dilute the stock to the required treatment concentrations. The SCC-9 cells were treated with fraction concentrations of 10, 20, 40, 80, 160, 320 and 640 μg/ml and media containing 1% DMSO was used as control (Keshava et al., 2016). 

*Cytotoxic effect of fractions on SCC-9 cells*

The SCC-9 cells were seeded in sterile flat bottom 96 -well plates at a density of 5 × 10^4^ cells per well and incubated for 24 h. After 24 hours, these cells were treated with fractions to final concentrations of 10, 20, 40, 80, 160, 320 and 640 µg/ml. The control cells were treated with media containing 1% DMSO. Following 24 hours of treatment, MTT assay was performed as described in Mosmann, 1983. The absorbance was measured at 590 nm using a microtiter plate reader (TECAN Spectra Fluor plus, MTX Lab Systems, Inc., VA, U.S.A.). The percentage growth inhibition was calculated using the following formula: Percentage inhibition of cell proliferation = [(Absorbance of control group-Absorbance of treated experimental group) / (Absorbance of control group)] ×100.

*Estimation of SCC-9 cells at various stages of cell cycle after treatment with ICLH fraction*

Approximately 1×10^6^ SCC-9 cells were cultured in a 6-well plate containing 2 ml of complete DMEM plus 10% FBS and incubated. After Thirty-two hours, the cells were serum starved by replacing media with DMEM containing 1% serum and incubated for an additional 32 h to synchronize the cell cycle. After cell cycle synchronization, a final concentration of 320 and 640 µg/ml of ICL extracts were added to the cells and only media was added to control cells. After 24 h of treatment, the cells were further processed as described previously in Keshava et al., 2016. The cells were analyzed for cell cycle distribution using BD FACS Calibur flow cytometer (Becton Dickinson, San Jose, CA, USA). The relative proportions of cells at the various phases of the cell cycle were estimated based on their DNA content, using an inbuilt CellQuest Pro software.

*Annexin V-FITC/propidium iodide dual stain assay for quantification of apoptosis in SCC-9 cells treated with ICLH fraction*

About, 1×10^6^ SCC-9 cells per well were incubated in serum containing medium with different concentrations (320 and 640 µg/ml) of I. cylindrica ICLH extract for 24 h in a CO2 incubator in a 6 well plate. The cells were subsequently stained with recommended concentrations of AnnexinV-FITC stain with PI stain and analyzed by flow cytometry using BD FACS Calibur (Becton Dickinson, USA) using excitation (λex)/emission (λem) of 488/520 nm for Annexin V-FITC and 540/630 nm for propidium iodide and analyzed by CellQuestTM Pro software. DMSO treated cells were used as control (Kuriakose et al., 2014).

*Investigating Caspase-3 and Caspase-8 Genes Expression in SCC-9 cells on treatment with ICLH fraction*

In order to investigate expression of Caspase-3 and Caspase-8 genes, Reverse transcription polymerase chain reaction (RT-PCR) was performed on synthesized cDNAs, using mRNA of SCC-9 cells. GAPDH was used as the internal control.

RNA Extraction and cDNA Synthesis. The cells were transferred to RNase-free microtubes. Total RNA was extracted using Trizol (Invitrogen) according to the manufacturer’s protocol. Purified RNA was then dissolved in 25 µl DEPC treated water. Prior to cDNA synthesis, RNA was quantified using a nanodrop UV spectrophotometer (ThermoFisher Scientific). The cDNA was synthesized from 2 μg of RNA using Verso cDNA synthesis kit (ThermoFisher Scientific) with oligo dT primer according to the manufacturer’s instructions. The reaction volume was set to 20 µl and cDNA synthesis was performed at 42^o^C for 60 min, followed by RT inactivation at 85^o^C for 5 min.

*RT-PCR*

The RT-PCR was carried out using appropriate forward and reverse primers obtained from Euro fins Genomics, India. The details of the primers and the expected size of the products of amplification are given in Table 1. The PCR mixture (final volume of 20 µl) contained 1 µl of cDNA, 10 µl of Red Taq Master Mix 2x (Amplicon) and 1µM of each complementary forward and reverse primers specific for Caspase 3, Caspase 8 and GAPDH and 6µl of distilled, deionised, sterile water was added to make the final volume of 20µl and MgCl_2_ concentrations of 1.5mM. Then the PCR was carried out under the following conditions: initial denaturation at 94^o^C for 4 min, followed by 35 amplification cycles, each consisting of denaturation at 94^o^C for 30 sec, annealing at 58^o^C for 30 sec, and extension at 7^o^C for 30 sec, with an additional extension step at the end of the procedure at 72^o^C for 5 min. Quantification of the results was accomplished by using Image J software. The values were normalized to the intensity levels of the internal control GAPDH (Amirkhiz et al., 2013).

*Statistical analysis*

All experiments were conducted in triplicates and the data was statistically analyzed using Graph Pad Prism (Graph Pad Software Inc. San Diego, USA). The MTT, apoptosis and cell cycle data were expressed as mean ± standard deviation (SD). Nonlinear regression analysis was employed to obtain dose-response curve on a logarithmic scale and the relative IC_50_ value was determined. The statistical comparison to determine significance of differences between treated and control group was performed by one-way or two-way ANOVA followed by Dunnett’s post-hoc test. The differences between the treated and control groups were considered significant when p<0.05. The gene expression data were analyzed using Kruskal-Wallis test and a p<0.05 was considered as significant.

## Results

*Cytotoxic effect of ICLH fraction on SCC-9 cells*

**Figure 1:**
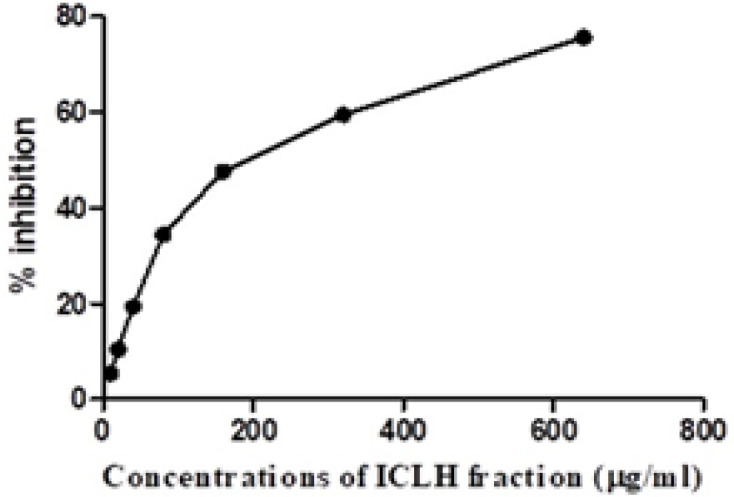
Cytotoxicity of ICLH Fraction on Human Tongue Squamous Cell Carcinoma (SCC-9) Cells Determined by MTT Assay. Dose-dependent growth inhibitory effect were observed in ICLH fraction treated SCC-9 cell line with a relative IC50 of 141 μg/ml. Inhibitory effect was not observed in the control cells

SCC-9 cells were subjected to cytotoxicity assay with all the three fractions of ICL methanolic extracts, i.e., ICLH, ICLC and ICLM fractions. However, effective cytotoxicity was observed only with the ICLH fraction. The highest cytotoxicity was observed at the higher concentrations of 320 and 640 μg/ml, and a percentage inhibition of cell proliferation was observed to be 59.47% and 75.65% respectively (Figure 1). A dose-dependent inhibition of the SCC-9 proliferation was observed after treatment with various concentrations of the ICLH fraction (Figure 1). The relative IC50 was determined to be 141 μg/ml for the ICLH fraction. The statistical analysis of the data was found to be significant with p value < 0.0001 (Figure 1). Since the highest inhibition of cell proliferation was shown at 320 and 640 μg/ml, further experiments were performed with these two concentrations of ICLH fraction. 

*Cell cycle inhibitory activity of ICLH fraction*

**Figure 2:**
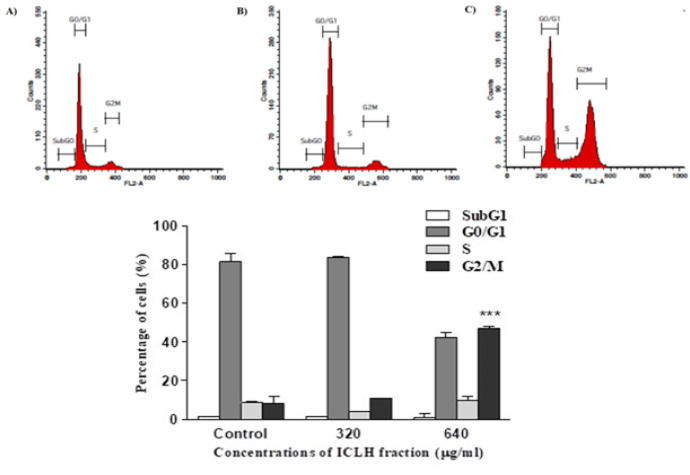
(Top) Representative flow cytometry plots. SCC-9 cells treated with media containing 1 % DMSO (control) (A); SCC-9 cells treated with 320 µg/ml (B) and 640 µg/ml (C) of ICLH fraction of ICL extract; (Bottom) Flow Cytometric Analysis of ICLH Fraction Treated SCC-9 Cells at Various Stages of the Cell Cycle. The percentage of cells in various phases of the cell cycle in both treated and control are indicated. The results are the mean ± SD of data from three independent experiments. When compared to control the results were statistically significant (***p<0.0001) at 640 μg/ml concentration of ICLH

Cell cycle inhibitory activity of the ICLH fraction against the SCC-9 cells was analyzed at 320 and 640 μg/ml concentrations (Figure 2). The percentage of cells in the various phases of the cell cycle in the control was observed to be 1.27%, 81.29%, 8.56% and 8.17% respectively for G0, G0/G1, S and the G2/M phases of the cell cycle. SCC-9 cells that were treated with 320 μg/ml of ICLH fraction showed highest percentage of cells, 83.7% at G0/G1 phase of the cell cycle. Whereas 1.86%, 3.92% and 10.96% cells were found in the G0, S and G2/M phases respectively. However, the statistical analysis of the data showed that the differences between the SCC-9 cells treated with 320 μg/ml of ICLH fraction and control were not significant.

The treatment of 640 μg/ml of ICLH fraction was observed to be more effective in terms of its cell cycle inhibitory activity. Treatment at this concentration resulted in accumulation of cells in both the G0/G1 and the G2/M phases of the cell cycle, at a percentage of 42.4 and 46.75 % respectively. The percentage of cells accumulated in the G0 and S phases were 0.99% and 9.84% respectively. All percentages given are an average value of triplicate experiments. The statistical analysis of the data observed for the treatment at 640 μg/ml as compared to the control was statistically significant with p value < 0.0001 (Figure 2). 

*Apoptosis inducing activity of ICLH fraction*

**Figure 3:**
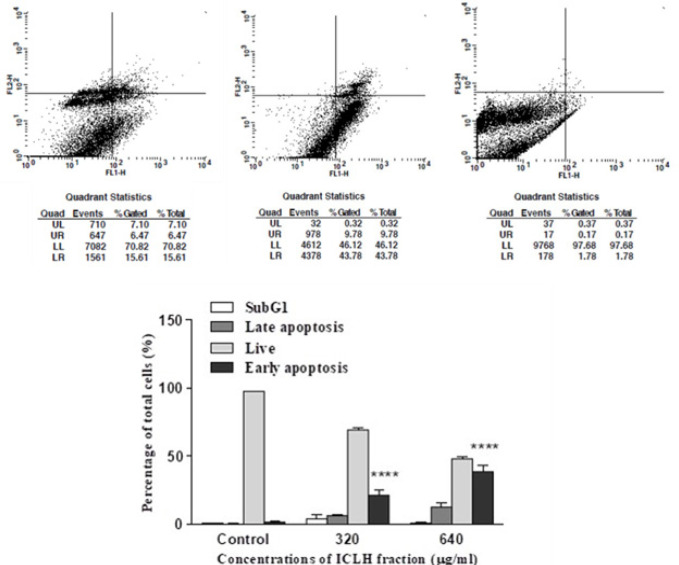
(Top) Representative flow cytometry plots depicting apoptosis in SCC-9 cells as observed in Annexin V-FITC/propidium iodide dual stain assay. (Left Panel) SCC-9 cells treated with 320 µg/ml of ICLH fraction; (Middle Panel) SCC-9 cells treated with 640 µg/ml of ICLH fraction; (Right panel) SCC-9 cells treated with media containing 1 % DMSO (control). Quadrants – Upper Left (UL) -Dead cells; Upper Right (UR) – Late apoptosis; Lower Left (LL) – Live cells; Lower Right (LR) – Early apoptosis: (Bottom) Flow Cytometric Analysis of ICLH Fraction Treated SCC-9 Cells undergoing apoptosis as observed by Annexin V-FITC/propidium iodide dual stain assay. The percentage of cells in late and early apoptosis in both treated and control are depicted. The results are the mean ± SD of data from three independent experiments. When compared to control the results were statistically significant (****p<0.0001) at 320 and 640 μg/ml concentration of ICLH

The percentage of SCC-9 cells undergoing apoptosis was evaluated at 320 and 640 μg/ml of ICLH fraction (Figure 3). Percentage of SCC-9 cells undergoing early apoptosis was observed to be 20.9% and 38.8% for 320 and 640 μg/ml of concentrations respectively. Percentage of cells in the late apoptosis stage was observed to be 6.16% and 12.49% at 320 and 640 μg/ml concentrations respectively. Whereas, in the DMSO treated control, the percentage of cells in early and late apoptosis were 1.58% and 0.24% respectively. All percentages given are an average value of triplicate experiments. Statistical analysis of the results of both the treatments were found to be significant compared to the control, with p value < 0.0001 (Figure 3).

*Effect of ICLH fraction on Caspase 3 and Caspase 8 expression in SCC-9 cells*

**Figure 4:**
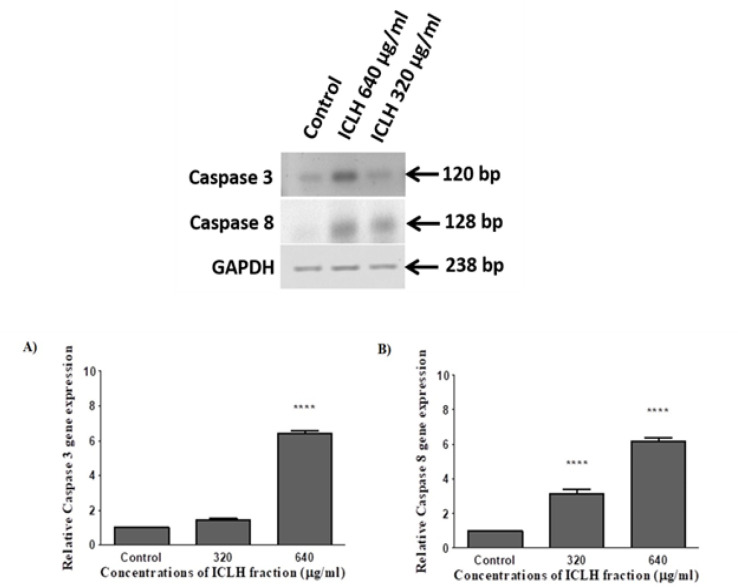
(Top) RT-PCR analysis of gene expression. Agarose gel electrophoresis of amplification products of Caspase 3, Caspase 8 and internal control GAPDH cDNA; (Bottom) Statistical analysis of Caspase 3 and Caspase 8 gene expression data. (A) Fold increase in caspase 3 gene expression in SCC-9 cells treated with 640 μg/ml of ICLH compared to control was significant (****p < 0.0036). (B) Fold increase in caspase 8 gene expression in SCC-9 cells for both 320 and 640 μg/ml of ICLH treatments, were significant (****p < 0.0036) compared to control

Induction of apoptosis was evaluated at the level of gene expression (Figure 4). Hence, the effect of ICLH fraction treatments on the gene expression of key downstream apoptosis executioner caspases 3 and 8 was analyzed. The gene expression was studied for treatments with the two concentrations, 320 and 640 μg/ml of ICLH fraction. SCC-9 cells treated with 320 μg/ml of fraction showed 1.46-fold increase in caspase 3 expression and a 3.16-fold increase in caspase 8 expression compared to the control. Whereas treatment with 640 μg/ml of ICLH fraction extract showed 6.45-fold increase in caspase 3 expression and 6.16-fold increase in caspase 8 expression as compared to the control. Statistical analysis of the data for caspase 8 expression revealed that the fold increase in the gene expression in SCC-9 cells, as compared to the control were significant with p<0.0036, at both 320 and 640 μg/ml ICLH concentrations, whereas, the fold increase in caspase 3 expression was significant (p < 0.0036) only at 640 μg/ml ICLH concentrations (Figure 4). 

## Discussion

Although successes have been achieved in chemotherapeutics, the systemic side effects caused by them is yet a matter of concern and hence the efforts to develop novel, alternative therapeutics is ongoing (Zhang et al., 2018). Several natural products have been tested against various OC cell lines. Apoptosis inducing effect of several medicinal plants in OC cells KB and ORL-48 have been reported (Majid et al., 2014). Withaferin A, a bioactive compound derived from a medicinal plant *Withania somnifera* has been reported to induce apoptosis in oral cancer cell lines HSC-3 and HSC-4 (Yang et al., 2015). Tenuifolide B from *Cinnamomum tenuifolium* stem is shown to inhibit cell proliferation and induce apoptosis in oral cancer cell lines Ca9-22 and CAL27 (Chen et al., 2016). Induction of apoptotic cell death has been reported in oral carcinoma cell line (KB) treated with *Myrmecodia tuberosa* extracts (Yuletnawati et al., 2016). 

In our previous study, we evaluated the anticancer properties of the methanolic extract of* I. cylindrica* leaves (ICL) using an oral squamous cell carcinoma cell line SCC-9 as an in vitro model system. The ICL methanolic extract caused cytotoxicity and induced cell death in SCC-9 cells in a dose-dependent manner. This treatment also reduced the clonogenic potential significantly and inhibited cell proliferation by arresting the cell cycle in the G2/M phase. Furthermore, apoptosis inducing ability of the ICL methanolic extract was shown by DNA fragmentation. We also demonstrated that the cytotoxic activity of the ICL methanolic extract was specific to the SCC-9 cancer cells, but not to the normal mouse embryonic fibroblast cell line NIH/3T3. Overall our data indicated towards the possibility of deriving potent anticancer therapeutics from the ICL methanolic extract, particularly against human oral cancers (Keshava et al., 2016). In continuation with these findings, the present study was aimed at bioactivity guided fractionation of the ICL methanolic extract in order to isolate the components possessing the anticancer activity. 

ICL methanolic extract was fractionated into non-polar, semi polar and polar components, using standard solvents such as n-hexane, chloroform and methanol respectively (Shahraki et al., 2015). The highest cytotoxicity against SCC-9 cells was observed (Figure 1) with n-hexane fraction (ICLH) and thus it was selected for further analysis. The highly non-polar solvent n-hexane is commonly used to derive non-polar bioactives from plants and these derivatives consist of the lipids and hydrocarbons (Shahraki et al., 2015; Abouelela et al., 2018). Our present data is in agreement with these results as the n-hexane fraction (ICLH), consisting of the non-polar components had the most significant cytotoxic activity against SCC-9 cells (Figure 1). Cytotoxic and anticancer activity of several such non-polar compounds from plant extracts have been reported for many human cancers such as hepatocellular carcinoma (Abouelela et al., 2018), human breast adenocarcinoma, prostate adenocarcinoma, and colorectal carcinoma cells (Li et al., 2016).

A dose dependent inhibition of the SCC-9 proliferation was observed after treatment with various concentrations of ICLH fraction. The relative IC50 was determined to be 141 μg/ml with highest activity recorded at 640 μg/ml concentration (Figure 1). Similar studies with n-hexane extracts of various plants have shown to inhibit of cell proliferation. For example, antiproliferative activity of the n-hexane fraction of *Nigella sativa* was shown against ACHN metastatic cell lines and transformed cell line GP-293 (Shahraki et al., 2015); Extracts from *Clinacanthus nutans* was toxic against human lung carcinoma and hepatocellular carcinoma (Ng et al., 2017); *Strobilanthes crispa*, extracts acted against hepatocellular carcinoma and breast cancer cells (Koh et al., 2017). Cytotoxicity of n-hexane extracts of *Aleuropus logopoides*, a grass species has been reported in breast, colon and liver cancer cell lines (Saleh et al., 2019). The IC50 concentrations of n-hexane extracts of the various plants showing antiproliferative activity ranged from 24 to 2000 μg/ml (Saleh et al., 2019; Koh et al., 2017; Ng et al., 2017; Shahraki et al., 2015). 

*Aleuropus logopoides* n-hexane extracts have been reported to inhibit cell cycle in breast, colon and liver cancer cell lines. The cell cycle arrest is induced in the G0/G1 phase of the cell cycle (Saleh et al., 2019). Similar cell cycle inhibitory properties against leukemic, hepatocellular carcinoma, and breast cancer cell lines have been reported for several n-hexane extracts of various plants (Belayachi et al., 2014; Koh et al., 2017). In corroboration with these results, data from the present study showed significant induction of cell cycle arrest at both G0/G1 and G2/M phases of the cell cycle in SCC-9 cells treated with 640 μg/ml of ICLH fraction (Figure 2). 

The ICLH fraction showed statistically significant apoptosis inducing ability against SCC-9 cells (Figure 3) and it also significantly increased the expression of key downstream apoptosis executioner genes caspases 3 and 8 (Figure 4). The n-hexane extracts of *Retama monosperma*, induced apoptosis in leukemic cells (Belayachi et al., 2014). Studies performed with n-hexane extract of *Strobilanthes crispa*, showed significant caspase 8 activation in hepatocellular carcinoma cells (Koh et al., 2017); n-hexane extracts of *Clinacanthus nutans* also showed induction of apoptosis by significant upregulation of caspase 3/7, 8 and 9 in human lung carcinoma and hepatocellular carcinoma cell lines (Ng et al., 2017). 

Data from the present study clearly establish the potential of ICLH extract to inhibit cell proliferation and induce apoptosis in the SCC-9 cells. These data also corroborate with similar studies performed with n-hexane extracts of other plants (Belayachi et al., 2014; Shahraki et al., 2015; Koh et al., 2017; Ng et al., 2017) including other grass species (Saleh et al., 2019). The cell cycle arrest observed at both G0/G1 and the G2/M phases of the cell cycle suggest that the active components of the extract could be acting through the G0/G1 and G2/M checkpoint machinery. The cell cycle arrest at the G0/G1 and G2/M phases could be due to a p53-mediated mechanism (Kavitha et al., 2017). The observed increased expression of the caspase 3 and 8 in the SCC-9 cells clearly indicates the induction of apoptosis in the treated cells. Activation of caspase 3 can be mediated through both mitochondria mediated intrinsic pathway or Fas mediated extrinsic pathway. However, the intrinsic pathway involves recruitment of the caspase 9/caspase 3 cascade whereas the extrinsic pathway depends upon the caspase-8/caspase-3 cascade (Mackenzie and Clark, 2012). In the present study, the upregulation of the caspase-8 and caspase-3 indicates towards the activation of the extrinsic apoptotic pathway. However, further analysis is required to determine the involvement of the intrinsic pathway. It is conceivable that both intrinsic and extrinsic pathways could be activated (Ng et al., 2017) which could be responsible for the apoptosis observed in the SCC-9 cells treated with ICLH. Further the treatment of SCC-9 cells with ICLH could result in increased levels of ROS, thus activating oxidative stress-induced apoptosis (Ng et al., 2017; Kavitha et al., 2017).

A study from India demonstrated that OC is interrelated with low income. In many developing countries, including India, most of the population do not have access to a well-organized and well-regulated cancer care system. Diagnosis of OC often leads to high treatment expenditures and causes significant economic burden on the patients and their families. The high costs involved in treatment and care also make several treatment options unaffordable to many (Kuriakose, 2018). Overall, from the results of the present study it can be concluded that ICLH extract possesses potential anticancer compounds. The natural abundance of* I. cylindrica *and its wide geographic distribution could render it as one of the primary natural resources for obtaining effective anticancer therapeutics with minimal systemic side effects. The abundance of this plant resource could make it a more cost-effective alternative for the discovery of novel anticancer drugs. Although few data are available on the anticancer activities *I. cylindrica* (Kuete et al., 2011; Kuete et al., 2013; Keshava et al., 2016; Kwok et al., 2016; Ravi et al., 2018), fewer anticancer compounds have been characterized from it (Wang et al., 2018). Hence further analysis of the ICLH fraction obtained in this study could result in development of effective anticancer therapeutics.

*Authors’ contributions*

RK – initiation of the present study, planning and execution of experiments, data analysis, data interpretation and manuscript writing; NM - execution of experiments, statistical analysis and helpful inputs to manuscript writing; RG – planning and execution of experiments, data analysis, data interpretation and manuscript writing; All authors have read and approved the final manuscript.

**Table 1:** Specifications of the Primers Used in RT-PCR

Gene	Primer pair	Sequence (5'à3')	Product size (bp)	Reference
Caspase 3	FP RP	ACATGGCGTGTCATAAAATACC CACAAAGCGACTGGATGAAC	120	Lan et al., 2014
Caspase 8	FP RP	CATCCAGTCACTTTGCCAGA GCATCTGTTTCCCCATGTTT	128	Wang et al., 2017
GAPDH	FP RP	CGACCACTTTGTCAAGCTCA CCCCTCTTCAAGGGGTCTAC	238	Jaganathan et al., 2013
